# A qualitative exploration of the content and face validity of preference-based measures within the context of dementia

**DOI:** 10.1186/s12955-020-01425-w

**Published:** 2020-06-11

**Authors:** Lidia Engel, Jessica Bucholc, Cathrine Mihalopoulos, Brendan Mulhern, Julie Ratcliffe, Mark Yates, Lisa Hanna

**Affiliations:** 1 School of Health and Social Development, Deakin University, Geelong, Victoria Australia; 2 Murdoch Children’s Research Institute, Royal Children’s Hospital, Parkville, Victoria Australia; 3 Centre for Health Economics Research and Evaluation, University of Technology Sydney, Ultimo, New South Wales Australia; 4 Health and Social Care Economics Group, College of Nursing and Health Sciences, Flinders University, Adelaide, South Australia Australia; 5 Ballarat Health Services, Ballarat, Victoria Australia

**Keywords:** Dementia, Quality of life, Outcome measurement, Preference-based measures, Proxy

## Abstract

**Background:**

Assessing the cost-effectiveness of interventions for people with dementia, based on cost per quality-adjusted life years (QALYs) gained, requires that the measures used to derive QALYs are preference-based whilst also being valid, feasible to use, comprehensible and acceptable for people with dementia. The aim of this study was to assess the content and face validity of six preference-based measures (PBMs) within the context of dementia.

**Methods:**

Qualitative focus groups and interviews were conducted with community-dwelling individuals with mild dementia and carers of people with dementia. After exploring participants’ understanding of ‘quality of life’ (QoL), six PBMs were assessed for content and face validity: two measures assessing health-related QoL (EQ-5D-5L and AQoL-8D); two covering broader aspects of capability wellbeing and social care-related QoL (ICECAP-O and ASCOT); and two dementia-specific QoL measures (DEMQOL-U and AD-5D). A random mix of one health-related QoL measure, one wellbeing measure, and one dementia-specific measure was explored in each session. All sessions were audiotaped and transcribed verbatim. Data were analysed thematically.

**Results:**

Nine individuals with mild dementia and 17 carers of people with dementia participated across 4 focus groups and 10 interviews. Participants perceived 9 broad QoL domains as relevant to them: Activity, Autonomy, Cognition, Communication, Coping, Emotions, End-of-Life, Physical Functioning, and Relationships. These domains had limited overlap with the content of the six PBMs. Assessment of face validity was summarized into eight themes: (1) ambiguous questions, (2) double –barrelled questions, (3) difficult/abstract questions, (4) judgemental/confronting questions, (5) lack of relevance and comprehensiveness, (6) response options, (7) layout/format and (8) proxy-response. There was no clear preference for one of the six measures explored; participants identified advantages and disadvantages across all measures. Although particularly designed for individuals with dementia, dementia-specific QoL measures were not always favoured over non-specific measures.

**Conclusion:**

Given the shortcomings of PBMs identified in this study, further empirical comparative analyses are necessary to guide the selection of PBMs for future dementia research.

## Background

Dementia is a syndrome that is characterised by the impairment of brain function, which affects language, memory, perception, personality and cognitive skills [1]. While there are different types of dementia, Alzheimer’s disease is the most common and accounts for two-thirds of diagnoses worldwide [1]. Given that there is currently no cure, treatment options typically focus on slowing disease progression and managing symptoms. Worldwide, dementia is a growing concern, with approximately 46 million people currently diagnosed [2] and estimated total global costs of US$ 818 billion [3]. As the number of people affected by dementia is expected to rise with ageing populations, there is an urgent need to address the associated economic burden by ensuring that the best quality care is delivered as efficiently as possible.

Many healthcare reimbursement authorities internationally use economic evaluation to assist policy and reimbursement decisions, where costs and benefits of alternative interventions are compared [4]. Cost-utility analysis (CUA) is the most common type of economic evaluation and is recommended by many regulatory authorities, including the Pharmaceutical Benefits Advisory Committee and the Medical Services Advisory Committee in Australia [5, 6] and the National Institute for Health and Care Excellence in the United Kingdom (UK) [7]. In CUA, outcomes are most commonly measured using quality-adjusted life years (QALYs), a health outcome where length of life is adjusted by quality of life (QoL), using a single utility value that reflects people’s preference for living in different health states. These values are measured on a 0 (dead) to 1 (full health) scale [8] and are often obtained indirectly using preference-based measures (PBMs). PBMs are largely health-related QoL questionnaires, which could be generic (applicable to an entire population) or condition-specific, with the inclusion of a scoring algorithm allowing the calculation of utility values.

One of the fundamental challenges in generating cost-effectiveness evidence for interventions in dementia is selecting the most appropriate outcome measures [9, 10]. To date, generic PBMs have been more widely used, as they allow for comparison of cost-effectiveness across different health areas [6, 7]. This is the case in dementia, where a recent review found that the generic PBM EuroQol five dimension measure (EQ-5D) was most frequently used [11, 12]. However, despite evidence supporting its feasibility, reliability and validity for people with dementia [11, 13], previous evidence has also indicated that the EQ-5D may lack the ability to incorporate aspects of QoL important for people with dementia and their carers [14]. The Assessment of Quality of Life eight dimensions (AQoL-8D) measure is another generic PBM that was developed to address the coverage limitations of the EQ-5D related to psychosocial elements of health [15]. Although the shorter versions AQoL-4D and AQoL-8 have previously been applied in people with dementia [16, 17], no evidence exists about the use of the AQoL-8D in a dementia-related setting.

Despite the fact that generic PBMs differ greatly in their coverage of QoL domains, they generally focus on health-related aspects of QoL. Yet, a previous study has indicated that older adults often derive QoL benefits from interventions that are not directly related to health (e.g., improved safety and independence or greater dignity) [18]. In recent years, two new PBMs were developed that go beyond health: the ICEpop CAPability measure for Older people (ICECAP-O) [19] and the Adult Social Care Outcomes Toolkit (ASCOT) [20]. Compared with generic HRQoL measures, these instruments measure a person’s broader capability wellbeing and social care-related QoL. While the ASCOT can be used to derive ‘social-care QALYs’, as it is anchored on a scale of 0 ‘being dead’ to 1 ‘ideal SCRQoL state’ [20], the ICECAP-O does not have QALY properties, given that it was anchored on a ‘full capability’ to ‘no capability’ scale [19]. National funding agencies in the UK and the Netherlands advocate the use of these measures for economic evaluations of social care interventions [21, 22]. Yet, a study in the Netherlands, comparing the EQ-5D-3L, ASCOT and ICECAP-O, found that older adults reported limitations in coverage, wording and interpretation [23]. Overall, evidence around these two measures remains limited and only two further studies have examined the validity and reliability of the ICECAP-O in people with dementia (both focused on professional proxies completed by nursing professionals) [24, 25]. To date, no evidence exists on the validity of the ASCOT in people with dementia.

Recently, dementia-specific PBMs have been developed for use in economic evaluation, such as the Dementia Quality of Life Utility (DEMQOL-U) [26] derived from the Dementia Quality of Life measure (DEMQOL) [27], and the Alzheimer’s Disease Five Dimensions (AD-5D) [28, 29] derived from the Quality of Life in Alzheimer’s Disease (QOL-AD) [30]. The purported advantage of such measures is that they focus on aspects of QoL that are affected by dementia, thus improving their validity and sensitivity. However, while the QOL-AD and the DEMQOL are the two most frequently used QoL measures in clinical trials of interventions for dementia [31], evidence supporting their preference-based versions is currently limited given their recent development [11].

In addition to the availability of several outcome measures that focus on different aspects of QoL (health-related, dementia-specific, and broader wellbeing), there are also measurement difficulties associated with the collection of QoL data in dementia [32–34]. Problems with cognitive function may interfere with understanding and ability to remember relevant events, as well as making judgments, which is why proxy responses are often sought. Previous studies have demonstrated differences between self and proxy-reports [35–42], with lower levels of association between caregiver ratings and self-ratings for subjective states when compared to physical states. Anosognosia (i.e., unawareness of deficits), variations in the meaning of QoL and the importance of QoL domains are reasons behind the discrepancies observed [33, 34, 43, 44]. Judgements about what is important to QoL may also change in individuals with dementia as the condition progresses, where broader aspects of QoL, such as safety and comfort, become more important to people living with dementia in later stages [34].

Collectively, all these aspects may jeopardize reliability and validity of QoL data from people with dementia. Nevertheless, economic evaluation based on QALYs requires measures that are valid, feasible to use, comprehensible and acceptable for people with dementia. Using non-validated PBMs for the assessment of cost-effectiveness of healthcare interventions can result in suboptimal funding decisions [45]. Given the current paucity of evidence around the validity of PBMs within the context of dementia, particularly for some of the recently developed measures, the aim of this study was to use qualitative methods to explore the content and face validity of PBMs in people living with dementia. Content validity is the degree to which an instrument measures the construct(s) it purports to measure [46]. In other words, it refers to how accurately an instrument taps into the various aspects of the specific construct in question, which in this case refers to the domains of QoL to be measured [47]. Face validity is a sub-type of content validity and is concerned with how understandable, appropriate and relevant the items of a particular instrument are ‘on its face’ when respondents complete them [48, 49]. Content validity is often assessed by conducting qualitative interviews with the targeted group of people to explore their perspective and experience on issues of importance to the measurement construct(s) [47], whereas face validity frequently involves cognitive debriefing exercises by actively testing the questionnaire items for relevance and interpretation [49]. The reliance on proxy-report, which becomes inevitable as the condition progresses, further implies that such validity examinations need to consider the views of caregivers to explore additional proxy-related challenges in administering these measures. Using a detailed examination of the perceptions of people with dementia and carers of people with dementia towards PBMs, this qualitative exploration of PBM aimed to provide further evidence on their validity for use in the economic evaluation of dementia interventions.

## Methods

The report of this qualitative study followed the Consolidated Criteria for Reporting Qualitative Research [50] and adheres to the dementia language guidelines published by Dementia Australia [51].

### Study design

Qualitative focus groups and interviews were carried out between August and December 2017 to explore the face and content validity of PBMs. Focus groups consisted either of people with dementia or of carers of people with dementia. Individual interviews were used complementarily, allowing the research team to accommodate participants who would have been otherwise unable to participate in the study due to travel distance, caring responsibilities, or being uncomfortable with focus group participation.

### Recruitment and consent

This study recruited people living with dementia and carers of people living with dementia (not necessarily dyads) within the Greater Melbourne region of Victoria, Australia. Dementia Australia, Carers Victoria, the Florey Institute, and the Cognitive Dementia and Memory Service (CDAMS) clinics advertised information about the study using newsletters, social media and flyers. Interested potential participants were directed to contact the study team who completed an eligibility screening over the phone. Community-dwelling people who self-reported a diagnosis of mild dementia, were 60 years and above, and were able to speak and read English were eligible to participate. Level of cognition was assessed via the 11-item telephone interview of cognitive status (TICS) [52]. Individuals with a TICS score above 21, indicating mild dementia, were eligible to participate. Eligible carers included individuals aged 18 or older who were able to speak and read English and who currently, or recently, had provided unpaid care to a family member or a friend with dementia of any severity. Furthermore, carers had to provide help for at least one activity of daily living, as defined by Lawton’s instrumental activities of daily living (IADL) scale [53]. To ensure that the person with dementia made an ‘informed decision’, the team asked the person with dementia to summarize what the study will involve and name the risks associated with this study. Following these additional steps, it was abstained from obtaining a proxy-informed consent from a legal guardian. Participants who met the eligibility criteria and agreed to participate were provided with an information sheet and consent form via email or post and offered dates and times for focus groups or interviews. On the day of the focus group/interview, all participants provided signed consent. Ethics approval for this study was obtained from Deakin University Human Research Ethics Committee (2017–103).

### Measures

Six measures were included in this study. These were the EQ-5D-5L, AQoL-8D, ASCOT, ICECAP-O, DEMQOL-U and QOL-AD. Table 1 describes each measure. The EQ-5D-5 L (Australian version) [54] was included because of its frequent use [11, 12]. The AQoL-8D is an Australian-based PBM [15, 56], which was included as it has a strong focus on psychosocial domains. The ICECAP-O [19] and ASCOT [20] were included given that their focus on social care and capabilities is relevant to elderly populations [57, 58]. DEMQOL-U [26, 59] and AD-5D [28, 29] were considered because of their focus on dementia-specific issues. Since the administration of the DEMQOL and QOL-AD is required to derive the DEMQOL-U and AD-5D, this study explored all 28 items of DEMQOL and 13 items of QOL-AD. Self-report versions were used for consistency reasons because proxy-versions were not available for all measures. Carers were instructed to comment on these measures from the perspective of the person with dementia, considering that this study focused on the appropriateness of the measures for people diagnosed with dementia.

**Table 1:** Characteristics of the measures included in this study

Conceptual origin	Generic health-related QoL		Capability wellbeing and social care-related QoL		Dementia-specific QoL	
Measure	EQ-5D-5 L [54]	AQoL-8D [15]	ICECAP-O [55]	ASCOT [20]	DEMQOL [44] (to derive the DEMQOL-U) ^a^	QOL-AD [30] (to derived the AD-5D) ^b^
Items	5	35	5	9	28 + overall QoL	13
Dimensions ^c^	5 Mobility; Personal-care; Usual activities; Pain/discomfort; Anxiety/depression	8 (4) Independent living; (3) Pain; (3) Senses; (8) Mental health; (4) Happiness; (3) Coping; (7) Relationships; (3) Self-worth	5 Attachment; Security; Role; Enjoyment; Control	8 Control over daily life; Cleanliness and comfort; Food & drink; Safety; Social participation; Occupation; Accommodation cleanliness and comfort; Dignity	4 (5) Positive emotion; (8) Negative emotion; (6) Memory; (9) Daily activities	13 Physical; Energy; Mood; Living; Memory; Family; Marriage; Friends; Self as a whole; Chores around the house; Fun things; Money; Life as a whole
Content development	Domains based on expert opinion and literature review; labels identified in face-to-face interviews with convenience samples of lay respondents	Previous measure (AQoL-6D), review; focus groups with members of the public and mental health consumers and carers, psychometrics	In-depth interviews with older adults	Previous measure (OPUS), literature review	Review, expert opinion, qualitative interviews, psychometrics	Literature review, interviews with people with Alzheimer’s Disease and caregivers, older adults, and experts

^a^Five items of the DEMQOL (#1, #4, #8, #14, #24) are used to derive the DEMQOL-U classification system (positive emotion, cognition, relationships, negative emotion, loneliness)

^b^Five items of the QOL-AD (#1, #3, #4, #5, #11) are used to derive the AD-5D classification system (memory, mood, living situation, physical health and do fun things)

^c^Each dimension is measured by one item except for the AQoL-8D and DEMQOL, which contain multiple items per dimension (presented in brackets)

### Procedure

Interviews were conducted and focus groups facilitated by LH (a female Associate Professor) and LE (a female Postdoctoral Research Fellow), with an observer (JB or LE) acting as note-taker. Focus groups and the majority of interviews took place at a University campus; some interviewees elected to participate at their home or other public place.

A semi-structured topic guide was used with open-ended questions supplemented with probes, where necessary, to enable responsiveness of participants’ emerging accounts and perspectives. Each interview/focus group consisted of two parts. In the first part, participants were asked to reflect on their understanding of the term QoL and factors influencing it, including the impact of dementia. Carers were encouraged to reflect on their experience of caring for someone living with dementia and how dementia affected the QoL of the person living with dementia. Data retrieved from the first part was used to generate domains of QoL that are relevant and important to participants in order to determine their inclusion or omission in existing PBMs to assess content validity.

The second part of the interview/focus group comprised a cognitive debriefing exercise in order to assess the face validity of PBMs. Participants were provided with copies of three of the six PBMs to minimize burden. These included one measure of HRQoL (EQ-5D-5 L or AQoL-8D), one measure of broader wellbeing (ICECAP-O or ASCOT), and one dementia-specific measure (DEMQOL or QOL-AD). In each interview/focus group, participants explored whether selected measures’ items and responses were appropriate and acceptable, interpreted accurately and relevant to participants’ lived experiences. Participants were asked the following questions: (1) What are your immediate thoughts about this questionnaire? (2) Is the wording of questions and response options clear? (3) Do you think this questionnaire is applicable to people living with dementia? (4) Is it comprehensive enough? (5) Are there any aspects of QoL missing? (6) Do you find it easy to complete? Carers were asked to reflect on how the person they care for would answer these questions and the feasibility of completing these measures on behalf of the person with dementia.

The sequence of measures was varied between the interviews to account for ordering effects and each measure was explored seven times across the interviews/focus groups. Carers were, generally, given a combination of measures that resulted in more items (average number of items discussed was 52 [min = 39; max = 73]) compared with measures that were discussed with people living with dementia (average number of items discussed was 44 [min = 27; max = 69]). Measures were not shared with any study participants prior to the sessions. Focus groups lasted for 90 min, whereas interviews took between 60 and 80 min. In each interview and focus group, the first 20 to 30 min were spent on part one. Most of the time was spent on the second part of the interview, i.e., the exploration of the PBMs. All sessions were audiotaped and transcribed verbatim prior to analysis. At the beginning of each interview/focus group, participants were asked to complete a short anonymous demographic questionnaire. All study participants were given a $30 gift card upon completion of the interview as a thank you for their time and participation.

### Data analysis

Transcribed interviews were first imported into NVivo 11 to facilitate data coding and retrieval [60], and analysed thematically [61]. Thematic analysis consisted of the following stages: familiarisation with the data (reading the transcripts); generating initial codes (organizing data into meaningful groups); searching for themes (sorting the codes into potential themes); reviewing themes (refining themes); defining and naming themes (development of a thematic map of the data and description of the content of each theme) [61]. Data obtained from both parts of the interview were analysed separately.

The thematic analysis of part one focused on the identification of QoL domains that were perceived as relevant to study participants. To assess content validity, these domains were then compared with the content of the PBMs. The second thematic analysis, for part two, summarized the comments made by study participants for each of the six PBMs to evaluate the face validity. Data analysis was carried out by LE, LH and JB and involved regular discussions with the research team.

## Results

In total, 9 individuals with mild dementia and 17 carers of people with dementia (five were dyads) participated across 10 interviews (*n* = 4 carers; *n* = 6 people with dementia) and 4 focus groups (*n* = 3 focus groups consisting of carers; *n* = 1 focus group with people with dementia). Characteristics of the study participants are provided in Table 2.

**Table 2:** Characteristics of study participants

	Person with dementia (*N* = 9)	Carer (*N* = 17)
Gender, Female (%)	4 (44)	12 (71)
Mean age (min; max)	74.9 (62; 84)	68 (52; 87)
Type of dementia (%)		
Alzheimer’s Disease	8 (89)	11 (65)
Younger Onset Dementia	1 (11)	–
Vascular Dementia	–	1 (6)
Lewy Body Disease	–	2 (12)
Frontal Lobe Dementia	–	1 (6)
Mixed (Alzheimer’s & Vascular)	–	2 (12)
Mean TICS score (min; max)	31 (22; 36)	–
Carer-reported severity level of dementia (%)		
Mild	–	3 (18)
Moderate	–	8 (47)
Severe	–	6 (35)
Years since diagnosis (%)		
Half to 1 year	2 (22)	0
1–2 years	4 (44)	1 (6)
2–3 years	2 (22)	1 (6)
3–4 years	0	3 (18)
More than 4 years	1 (11)	12 (71)
Living arrangement of person with dementia (%)		
Living alone	1 (11)	2 (12)
Living with (a) family member(s)	7 (78)	8 (47)
Living in a care institution	–	4 (24)
Deceased	–	3 (18)
Other	1 (11)	–
Relationship to person with dementia (%)		
Partner	–	10 (59)
Daughter/Son	–	6 (35)
Another family member	–	1 (6)
Frequency of providing informal care (%)		
Daily	–	14 (82)
3–6 times per week	–	3 (18)

Among the people with dementia, Alzheimer’s Disease was the most common type of dementia, with 44% being diagnosed 1–2 years ago. Most carers were female (71%) and cared for their partner (59%) who most commonly had Alzheimer’s Disease (65%), although other types of dementia were also reported. Carers provided informal care on a daily basis (82%), which reflected their shared living situation (47%). However, 4 carers (24%) provided informal care to a person with dementia residing in a care institution, 2 carers (12%) for a person with dementia who was living alone and 3 carers (18%) reported that the care recipient had passed away.

### Content validity

Study participants identified a range of aspects of QoL that they perceived as important. These were thematically summarized into nine domains: Activity, Autonomy, Cognition, Communication, Coping, Emotions, End-of-Life, Physical Functioning, and Relationships.

#### Activity

The activity domain refers to the day-to-day life of people living with dementia and the desire to undertake enjoyable and meaningful activities. It describes the: “*…satisfaction with what you’re doing, that you have things that gives you a sense of being, a sense of doing something and being actively engaged”* (PWD4). It ultimately emphasized the importance of “…*being purposeful*” (PWD4). Study participants also mentioned the small things in life that bring them joy. One study participant said: “*I can still have a drink of red wine. I can go to the races. I’m just enjoying myself*” (PWD1). In addition to doing things on their own, engaging with the community was also important: “*So that community is incredibly important to us. But then also networks within the local community, like simple as it sounds, him independently meeting people at the coffee shop*” (Carer, FG2).

#### Autonomy

Retaining one’s own identity and autonomy was key to maintaining good QoL. In particular, carers discussed the importance of a sense of control and independence, which people living with dementia are often unable to uphold as the condition progresses: “*If somebody starts to lose their control, whether it’s over physical or mental or any other capacity – of course, that greatly affects their quality of life*” (Carer, FG1). Carers often witnessed firsthand when the person living with dementia starts to lose certain abilities, and made comparisons between the person’s past and current levels of independence: “*Mum’s always been an independent person […] from the banking to the shopping, decision making, living on her own, not accepting help from others, providing the help to others, being there. […] It’s now at the stage where this independence comes down to things like toileting*” (Carer, FG2).

#### Cognition

Dementia has a large impact on a person’s cognition. However, different aspects of cognition can be affected and were mainly discussed by carers of people living with dementia. Alongside a lack of concentration, where “…*your thoughts are muddled*” (Carer2), there is also often confusion. One carer described: “…*she’d get very confused in areas that she wasn’t familiar with. And on a number of occasions, would actually say to me: Who is that person sitting next to me?*” (Carer, FG4). People living with dementia also talked about their forgetfulness and how it made them feel: “*Well – being forgetful means that I just forget, and that’s tough. I wish I didn’t forget*” (PWD3). While people in the later stages of dementia often lack awareness and insight, carers described that in early stages “*They’re very aware of their memory. But they’re also aware that they forget everything*” (Carer, FG4).

#### Communication

Besides cognition, dementia also affects people’s communication skills and their ability to express themselves verbally. This often causes feelings of frustration as a carer described: “*At the moment now she’s also losing her words and there’s a frustration in her with verbally not being able to communicate what she feels*” (Carer, FG2). As a consequence of this, people living with dementia may not be understood by others: “*Well, he will answer the phone, but then he says all these silly things, muddy things. The person at the other end doesn’t know what’s going on*” (Carer, FG4). On the other hand, people living with dementia also have difficulties understanding other people, especially in a crowded environment: “*If there’s a lot of people talking, I wouldn’t pick it up*” (PWD, FG3).

#### Coping

To pursue a good life despite dementia, carers and people with dementia discussed different ways to cope with dementia. A key mechanism to adapt/adjust to the condition was to create structure in day-to-day life: “*We have to run a very disciplined diary […] I’ve lost a lot of the capacity to plan things or I forget them*” (PWD4). People living with dementia often recognized that they were losing their abilities and level of independence and a further coping approach discussed was acceptance of the situation: “*Yes, she’s [wife] taken over all the finances. I used to run all our finances…Well, it wasn’t okay for me but it was something that was necessary*” (PWD4). Others, however, would withdraw “*because [they] don’t want to be ineffective with people, or do things that are not loving towards people*” (PWD3). Carers observed that the person living with dementia was in denial: “…*often enough she’ll blame me, often enough she will deny that something she’s been told, or something that she’s been made aware of, and that is a bit frustrating*” (Carer4). Some people living with dementia had held onto their religion or spiritual beliefs to cope with their situation: “*This is where I get religious, God is good, and God will look after me, and there’ll be lots of fulfilling things happen anyhow, so I’ll just go with it*” (PWD3).

#### Emotions

Different emotions were discussed, reflecting the experiences of people living with dementia that were both positive and negative. While people living with dementia talked about the various things that brought them joy and happiness in life, carers perceived that their role was to “…*trying to keep [them] happy”* (Carer3). With the loss of independence and cognitive decline, people living with dementia often experienced anxiety and feelings of frustration: *“She doesn’t know where the toilet is. That then brings on an anxiety and behavioural issues”* (Carer, FG2). Especially in the early stages of dementia, awareness of their forgetfulness frustrated people living with dementia: “*Well, I was forgetting things, that was one of my major problems which was very frustrating for me, and probably for my husband as well, because I’ll ask him a question and then half an hour later, I’ll ask him the same question”* (PWD6).

#### End-of life

Receiving a diagnosis of dementia meant for many people with dementia that they had to make important end-of-life decisions: “*I think I’m confronted by my own death more than I have before […] I’m confronted with my mortality through this diagnosis*” (PWD3). However, often a diagnosis had been made too late and had left people living with dementia with fewer choices: “*I feel if he had a proper diagnosis before then…there probably would have been more choices*” (Carer2). People living with dementia were not only confronted with their own death but were also concerned about the future of their loved ones: “*The only thing that distresses me about it is the thought that I am going to die and she is going to be left*” (PWD 5). Carers of people living with dementia living in a residential aged care facility also discussed the importance of the environment when nearing end-of-life: “*I really came to the conclusion that the mission of anybody who’s caring for people with dementia is to help them to come to their end-of-life, and that does not happen in the residential facilities. People just die anonymously […] They just go on and are forgotten*” (Carer, FG1).

#### Physical functioning

Staying physically fit was discussed numerous times, as the key to maintaining a certain level of independence but also being able to undertake enjoyable activities: “*I’m able to get around; I can dance, I can walk, I walk my dog round the place every day, twice a day. So physically I am fit*” (PWD, FG3). As physical functioning declined, the ability to self-care reduced: “*My Mum still showers herself. Sometimes she doesn’t clean her teeth terribly thoroughly and she has incontinence and wears pads*” (Carer, FG2).

#### Relationships

Having meaningful interactions with family and friends was considered an important aspect of quality of life: “*Oh, I think it’s very important, and a good family too, especially the grandchildren, I enjoy seeing them*” (PWD 6). Carers also perceived that their role was to maintain the levels of quality of life of the care recipient: “*Well, if I wasn’t there, his quality of life would be awful. He would’ve burnt the house down, sort of thing. He does need someone there - so I try to keep his quality of life*” (Carer, FG4). In this context, carers also talked about the importance of treating the person with dementia with respect and dignity: “*I think a sense of self-worth, a sense of quality of life, is respect and being treated as an adult who has contributed to society*” (Carer, FG1). With the greater reliance on carers, people living with dementia also expressed their concerns about being a burden to others: “*Well sometimes I feel, that it concerns me a little bit, if my dementia gets worse that would be a bit of a strain on him. That concerns me*” (PWD 6). People living with dementia also talked about their perceived level of acceptance by others, especially around their diagnosis: *“…because there’s so many people that are accepting. If I didn’t have a whole heap of people accepting me, then maybe it would be more important, but if a few fall off the boat at the moment because they can’t cope with my Alzheimer’s, well, that’s their problem*” (PWD3).

#### Comparison of QoL domains with the content of PBMs

A comparison of these domains and sub-domains with the content of the descriptive systems of the six existing PBMs revealed that the measures captured some but not all domains that were perceived as relevant (Table 3). All measures, except the DEMQOL-U, contain items related to *Activity* and *Physical Health*. While most measures also capture some aspects of positive and negative *Emotions* (with the exception of the ASCOT) and *Relationships* (except for the AD-5D), none of the measures contain items related to *End-of-life* aspects that ware identified as important aspects influencing QoL. Only the ICECAP-O asks about concern when thinking about the future, which overlaps with the end-of-life domain. *Coping* features only in the AQoL-8D. The AQoL-8D was also the only measure, besides the DEMQOL-U, that assesses *Communication* abilities. Not surprisingly, only the two dementia-specific PBMs contain items related to *Cognition*, but are also the only measures that do not contain items related to *Autonomy*, such as control or independence.

**Table 3:** Quality of Life domains and sub-domains identified as important and their inclusion in existing PBMs

Domain	Sub-domain	EQ-5D-5 L	AQoL-8D ^a^	ICECAP-O	ASCOT	AD-5D	DEMQOL-U
Activity	Community engagement	✔ (Usual activities)	✔ (Relationships & Happiness)	✔ (Enjoyment)	✔ (Occupation)	✔ (Do fun things)	
	Enjoying things						
	Purposeful activities						
Autonomy	Control	? (Self-care)	✔ (Coping & Independent living)	✔ (Control)	✔ (Control over daily life)		
	Independence						
Cognition	Awareness/ Insight					✔ (Memory)	✔ (Cognition)
	Concentration						
	Confusion						
	Forgetfulness						
Communication	Expressing yourself verbally		✔ (Senses)				? (Relationship)
	Being understood						
	Understanding						
Coping	Acceptance of situation		✔ (Coping)				
	Adapting/ adjusting						
	Denial						
	Religion/ spirituality						
	Withdrawal						
Emotions	Anxiety	✔ (Anxiety/Depression)	✔ (Mental Health & Happiness)	? (Enjoyment)		✔ (Mood)	✔ (Positive emotion and Negative emotion)
	Frustration/ agitation						
	Happiness						
End-of-life	Choice			? (Security)			
	Environment						
	Thinking about the future						
	Preparation for death						
Physical functioning	Physically fit	✔ (Mobility)	✔ (Independent living)	? (Control)	? (Cleanliness and comfort)	✔ (Physical health)	
	Self-care						
Relationships	Accepted by others		✔ (Relationships & Self-worth)	✔ (Attachment)	✔ (Social participation & Dignity)		✔ (Relationships & Loneliness)
	Burden to others						
	Carer						
	Family						
	Friendships						
	Relationship with spouse/partner						
	Treated with respect and dignity						

^a^ Inclusion of important quality of life domains by the respective domains of the AQoL-8D

### Face validity

A second thematic analysis was undertaken of participants’ comments when assessing the face validity of the measures. This analysis revealed eight themes: (1) ambiguous questions, (2) double –barrelled questions, (3) difficult/abstract questions, (4) judgemental/ confronting questions, (5) relevance and comprehensiveness, (6) response options, (7) layout/format, and (8) proxy-response. It should be noted that comments made on dementia-specific measures refer to the original measure and not their preference-based versions. Each of the eight themes is discussed in more detail below and illustrative quotes are presented in Table 4.

**Table 4:** Illustrative participant commentary on the face validity of the six PBMs

Measure	Item/section/overall measure	Illustrative participant commentary
**Ambiguous items**		
AQoL-8D	Item 3: ‘How easy or difficult is it for you to get around by yourself outside your place of residence (e.g.*. to go shopping, visiting*)?’	“How easy or difficult, are we saying what we actually use to get around, I need an aide or…?” (Carer1)
AQoL-8D	Item 7: ‘How much confidence do you have in yourself?’	“Confident to speak, confident to – I don’t know, what is it, confidence to do what?” (Carer1)
ICECAP-O	Item 2: ‘Thinking about the future’	“Well what kind of concern do you mean?” (FG3, PWD)
ICECAP-O	Item 3: ‘Doing things that make you feel valued’	“I sometimes think what do you mean by feel valued? What’s the value?” (FG3, PWD)
ICECAP-O	Item 5: ‘Independence’	“When you say being independent is that what you physically do, think or how you act or what?” (FG3, PWD)
QoL-AD	Item 12: ‘Money’	“In question 12, is that really asking me if I’ve got a lot of money or if I’m poor?” (PWD1)
**Double –barrelled questions**		
ASCOT	Item 5: ‘Thinking about how much contact you have with people you like, which of the following statements best describes your social situation?’	“Well say number five, just because it’s a double barrel request in that you’ve got to think about the way you think about people… So you’re required to have two concepts in your mind at once and interrelate them. And I think a number of those questions required you to do that.” (Carer, FG2)
AQoL-8D	Item 10: ‘How satisfying are your close relationships (family and friends)?’	“…some of the questions may need to be separated. Like, ‘How satisfying are your close relationships with family and friends?’ That could be two totally separate things.” (Carer, FG2)
EQ-5D-5L	Item 5: ‘Anxiety/Depression’	“Well, to me, anxiety and depression are two quite different things. I mean, they’re just sort of lumped together, isn’t it?” (Carer, FG4)
EQ-5D-5L	Item 4: ‘Pain/Discomfort’	“Good questionnaire, except the anxiety and depression. I think that should be separate. And maybe the pain and discomfort.” (Carer, FG4)
ICECAP-O	Item 1: Love and Friendship	“Well, I think possibly if it were two separate questions, I would’ve ticked four for: “I can have all of the love that I want.” Whereas I would tick three: “I can have a lot of the friendship that I want”.” (Carer, FG4)
**Difficult/abstract questions**		
ASCOT	Item 8: ‘Which of these statements best describes how having help to do things makes you think and feel about yourself?’	“So I’ve never thought of this, having help or - sorry, having help making me feel better about myself.” (PWD4) “I think someone with her level of dementia might find it difficult and would probably appreciate having the question expanded a bit more.” (Carer4)
ASCOT	Overall	“I found the questions difficult in some cases to understand and also I didn’t think that I would be able to describe them very easily to my husband.” (Carer, FG2)
AQoL-8D	Item 9: ‘Does your health affect your relationship with your family?’	“Oh my role in the family, my role in the family is something that you have to think about – what that meant.” (PWD5)
AQoL-8D	Item 8: ‘Do you normally feel calm and tranquil or agitated?’	“Well, I don’t know what you’re asking. Tranquil, I don’t know what it is.” (PWD1)
AQoL-8D	Item 17: ‘How enthusiastic do you feel?’	“Once again the word enthusiastic, what is enthusiastic?” (PWD1)
DEMQOL	Section 1: ‘Questions about your feelings’	“Well, what’s the difference between feeling lively and full of energy? That’s almost the same, isn’t it?” (FG4, C)
DEMQOL	Section 2: ‘Questions about your memory’ Section3: ‘Questions about your everyday life’	“The second and third lot just wouldn’t be answerable by my wife.” (Carer, FG1)
DEMQOL	Section 2: ‘Questions about your memory’	“Like, where my wife’s at – there’s no point asking anything about the memory. She can’t even repeat – she says a sentence to me, and I try and ask her to repeat it, and she can’t do that.” (Carer, FG1)
QoL-AD	Item 9: ‘Self as a whole’	“Self as a whole - What does that mean?” (PWD5)
**Judgemental/ confronting questions**		
ASCOT	Item 7: ‘Which of the following statements best describes how clean and comfortable your home is?’	“No, not offended, but you get defensive in your answers, and you’d be saying ‘I don’t want to give this impression’…– ‘of course my house is clean.” (Carer4)
DEMQOL	Item 21: ‘How you get on with people close to you?’	“…reword that because the minute you say, “How do you get on?” I think that’s a little bit loaded” (Carer, FG1)
DEMQOL	Item 24: ‘Making yourself understood?’	“I would reword that, too, and say: Do you feel others are understanding you well?” (Carer, FG1)
DEMQOL	Section 2: ‘Questions about your memory’	“My mother would definitely be on the defence, and she would give me a very generic answer to any of these questions…She would have been quickly into denial.” (Carer, FG1)
EQ-5D-5 L	Item 1: ‘Mobility’ Item 2: ‘Personal care’	“About the personal care – my mother would lie about it, for sure. The mobility, it’s another one.” (Carer, FG1).
ICECAP-O	Overall	Maybe this is a male-female thing, I don’t know, but I think my uncle would find this document [ICECAP-O] more confronting than the last one. (Carer, FG1)
**Relevance and comprehensiveness**		
ASCOT	Item 7: ‘Which of the following statements best describes how clean and comfortable your home is?’	“…it’s self-explanatory because most people would feel better if their house is clean, sort of thing” (PWD4). “And I wonder, this is almost a sexist way but I don’t mean it that way, where, traditionally, women who have kept house would feel more about that than other people might.” (PWD4)
ASCOT	Item 3: ‘Thinking about the food and drink you get, which of the following statements best describes your situation?’	“Well, I don’t think it’s terribly important. I mean I get very well fed here.” (PWD5)
AQoL-8D	Item 16: ‘Do you ever feel like hurting yourself?’	“I really can’t comment on that because I’ve never [thought of that].” (PWD1)
AQoL-8D	Item 6: ‘How often do you experience serious pain?’ Item 22: ‘How much pain or discomfort do you experience?’	“Didn’t we cover pain before? 6 and 22, I think, want to become one question.” (PWD1) “Is that a repeat – I can’t remember where it was, but it seems that that’s slightly repetitive to another one.” (PWD3)
AQoL-8D	Overall	I think something about being bored [is missing]. (Carer, FG2) Family, friends. Is there anything here about partners? (Carer1)
DEMQOL	Overall	“While I feel that these are important – feeling, memory and life – so, you’re definitely attacking the right avenue, but the whole thing is very generic.” (Carer, FG1)
DEMQOL	Section 2: ‘Questions about your memory’	“At the early stages of dementia, I think those would be relevant.” (Carer, FG1) “I don’t actually think these should be asked because he would feel offended.” (Carer, FG1)
DEMQOL	Section3: ‘Questions about your everyday life’	You could add a last question to the third part. Just some sort of general question about, “How organised do you feel you are?” or, “How easy is it to be organised in your life?” (Carer, FG1)
EQ-5D-5 L	Overall	“It’s a softy. It’s not very inquiring, I guess.” (PWD4) “This is more for carers than the actual person with dementia” (Carer, FG1). “This is a more objective.” (Carer, FG1) “This asks relevant questions. Because, if they’re aware they’re losing these capacities, then they’re in trouble. And I think asking these questions is really quite important.” (Carer, FG1)
ICECAP-O	Overall	“This is about subjective values.” (Carer, FG1) “They don’t touch much on emotions…“Do you feel sad?” “Do you feel upset,” “Do you feel depressed.” (PWD3)
ICECAP-O	Item 2: ‘Thinking about the future’	“Most of the time, they don’t have a future. They don’t want to think about it. Many times, I have certain questions for Mum, and she says, “I don’t care,” because they know they don’t have much of a future coming.” (Carer, FG1)
QOL-AD	Overall	“I think this one [AQoL-8D] is better because it does reflect on your mood, and everything else, and shows a bit more of the person, whereas you can hide behind this [QOL-AD]” (PWD5). “This [QOL-AD] would be a great snapshot that maybe could be asked regularly and then averaged out kind of thing. It’s just a touching point.” (Carer, FG2)
**Response options**		
ASCOT	Item 8 and item 9: ‘Having help sometimes undermines the way I think and feel about myself”	“She’d probably say ‘what do you mean by undermines?” (Carer4)
ASCOT	Overall	“Oh so the question is in the answer? Yes. And so I think for my mother that would make it easier for her to fill out.” (Carer, FG2)
ASCOT	Overall	“‘As much as you want’, ‘Adequate’ – more or less the same thing like, in my mind.” (Carer4)
AQoL-8D	Overall	“I thought the statements, ‘I have as much control,’ or ‘I have no control,’ [ASCOT responses] would be easier for my Mum to comprehend than ‘often’, ‘never’ or ‘most/sometimes’ [AQoL-8D responses].” (Carer, FG2)
AQoL-8D	Item 15: ‘I am very mobile’ and ‘I have no difficulty with mobility’	“Answer one and two are really the same thing aren’t they?” (PWD1)
AQoL-8D	Item 19:‘These things are very easy for me to do’ and ‘I have no real difficulty in doing these things’	“Answer one and two are the same to me.” (PWD1)
EQ-5D-5 L	Overall	“I would never put a ‘severe.’ They’re going to say, “I’m not severe”. I would always talk […] much softer. Give them an out.” (Carer, FG1)
ICECAP-O	Item 1: ‘Love and Friendship’	“Once again the first option and the second option are, they’re the same. ‘I can have all of the love and friendship’, or ‘I can have a lot of the love and friendship’?” (PWD1).
ICECAP-O	Item 1: ‘Love and Friendship’ Item 4: ‘Enjoyment and pleasure’	“I can have, does that mean I do have right now, or I have the ability to, or the capacity to?” (PWD3)
QOL-AD	Overall	“…because it makes it very open…it doesn’t push you in one way or another to frame - how you frame your answers” (PWD4).
**Layout and format (including instructions and recall time)**		
AQoL-8D	Overall	“…it would take a long time to do this questionnaire.” (Carer, FG2)
ASCOT	Overall	“I struggled with this one. I think it’s too many words.” (Carer, FG2)
ASCOT	Item 4 - instruction: ‘By ‘feeling safe’ we mean how safe you feel both inside and outside the home. This includes fear of abuse, falling or other physical harm.’	“I don’t think abuse would have even come onto the horizon” (Carer4) “Yeah, well, I jumped [the instructions], really, and [only considered safety] inside the house” (PWD4).
DEMQOL	Overall – recall time (last week)	“…in [her partner’s] case, he would be both cheerful and enjoying life as well as distressed and sad and lonely – all within a fairly short time frame?” (Carer 2)
DEMQOL	Overall	“It’s a bit more - because it’s so compact, it’s a bit more difficult. But in terms of the content, the content was fine, it’s just making sure that you kept everything in line.” (PWD2)
EQ-5D-5 L	Overall	“…it is the best format for someone with dementia.” (Carer, FG4)
**Proxy response**		
ASCOT	Overall	“I found the questions difficult in some cases to understand and also I didn’t think that I would be able to describe them very easily to my husband.” (Carer, FG2) “I could relate to how [her] Mum would answer them more easily...because they’re more general” (Carer, FG2).
AQoL-8D	Overall	“I would be able to manage this, no problem at all.” (Carer, FG1) “Frustrated, confident, full of energy - well, that’s a very subjective thing, isn’t it? Lonely, distressed…” (Carer, FG4)
DEMQOL	Overall	“I mean, you can also tell about the frustration or distress. But a lot of those things are very much subjective, and it’s difficult to honestly answer.” (Carer, FG4) ‘Questions about your memory’: “See, that’s easier to answer, as a carer answering on behalf of the person you’re caring for because you’re a witness to these things.” (Carer, FG4)
EQ-5D-5 L	Overall	“It could not be answered by the person with dementia, but it could be observed by the carer…. if she’s feeling any pain, because it looks uncomfortable.” (Carer, FG1)
ICECAP-O	Overall	“…if you look at thinking about the future... I’m only answering on the basis of what I think she’s thinking; not because she’s ever said to me, ‘I’m concerned about the future” (Carer, FG4).

*FG* Focus group, *PWD* Person with dementia

#### Ambiguous questions

Ambiguous questions refer to questions in which there is more than a single way to interpret it. Participants identified a number of ambiguous terms across multiple measures. Particularly questions in the ICECAP-O created ambiguous interpretation for people with dementia because certain terms, such as ‘independence’ were perceived as vague that could have more than one meaning (i.e., physical independence or mental state of freedom).

#### Double-barrelled questions

Study participants also discussed double-barrelled questions, which refer to questions that touch upon more than one issue, yet allow only for one answer. This was often an issue for composite questions, where two different concepts were merged into one question, such as relationships with friends and family (AQoL-8D), love and friendship (ICECAP-O), or the combination of anxiety and depression in the EQ-5D-5L. Carers also thought that the ASCOT contained a number of questions that “…*required to have two concepts in your mind at once and interrelate them”* (Carer, FG2), which could be challenging for people with dementia.

#### Difficult/abstract questions

Some questions were generally perceived as difficult if they required respondents to reflect in an abstract way on their lives. There were some words in the AQoL-8D that were not clearly understood by some study participants and also the dignity question of the ASCOT received mixed responses, where some participants stated that they have never thought about ‘how having help makes them think and feel about themselves’. With regard to the DEMQOL, some carers expressed concerns particularly regarding section 2 (memory) and section 3 (everyday life), which would be difficult to answer for people with dementia.

#### Judgemental/ confronting questions

A number of carers thought that some of the questions were judgmental, confronting or offensive. As a carer stated: “*Everything you say to them must be carefully vetted, in case it can be interpreted as judgemental”* (Carer, FG1). Numerous questions across measures appeared to be problematic, such as the ASCOT cleanliness and accommodation, where people with dementia may not want to give the impression that their house is not clean. As a potential explanation for their motivation to lie, one carer described that *“they didn’t want anyone to take them out of their own home, their own environment”* (Carer1). There was also a discussion around the importance of asking memory-related questions. Particularly for the DEMQOL, which contains a number of memory-related questions, carers felt that by asking such questions, the person with dementia would feel judged or offended, and will be immediately at the defence. Carers also talked about the person’s with dementia potential motivation to influence someone that was applicable to all measures: “…*even if they could understand okay, wouldn’t necessarily answer it at face value. It would be reflecting a range of other considerations about, for example, what they want to communicate or how they want to influence someone”* (Carer, FG2).

#### Relevance and comprehensiveness

The AQoL-8D was perceived as a comprehensive measure, covering a number of relevant questions, although study participants noted some repetition and irrelevant questions. Some irrelevant questions were also noted for the ASCOT (e.g., food and drink and accommodation cleanliness and comfort). While study participants thought that all questions of the QOL-AD were relevant, they cautioned at the same time that they would only provide a “*snapshot”* and should be interpreted as “*a touching point”* (Carer, FG2). A similar comment was made with regard to the EQ-5D-5 L, which was perceived as: “…*a softy. It’s not very inquiring, I guess.”* (PWD4). Compared with the EQ-5D-5 L, that was described as ‘*objective’*, the ICECAP-O was perceived to be about ‘*subjective issues’* (Carer, FG1), which covers a number of good and relevant questions. Mixed opinions were expressed towards the relevance of question 2 of the ICECAP-O (thinking about the future) and the relevance of the memory section of the DEMQOL. Participants also identified some domains that were missing, such as being bored’ and a question targeting the relationship with the partner in the AQoL-8D, questions related to negative emotions in the ICECAP-O, and a question about the ability to be organized in life in the DEMQOL.

#### Response options

There were great differences in terms of the format across the measures. Carers felt that measures presenting the question and response options as a series of statements (e.g. EQ-5D-5 L or ASCOT) were more appropriate for people with dementia. On the other hand, it also increases the amount of reading and some participants did not like lengthy response options. Response options that referred to frequencies, such as often, never, most of the time etc., included in the AQoL-8D, were generally perceived as more difficult to comprehend relative to response options embedded within questions*.* Several response options were also not mutually exclusive that were found in the AQoL-8D and the ICECAP-O.

#### Layout and format

The general layout and the length of the measures were also discussed. To be appropriate for use in individuals with dementia, carers emphasized that the measures need to be short. Otherwise, *“if it’s too long, they’ll get in a muddle”* (Carer, FG4). While the length of the EQ-5D-5 L was perceived as acceptable (“…*it is the best format for someone with dementia”* (Carer, FG4)), carers and people with dementia thought that the AQoL-8D was too long. In this context, the amount of reading was perceived as important, “…*as some people with dementia struggle with actually reading and interpreting what’s there”* (Carer, FG4). The amount of reading was particularly an issue for the ASCOT, which despite being a “*very short and pretty easy”* questionnaire (PWD2), contained “*too many words”* (Carer, FG2), which would be very challenging for a person with dementia. Furthermore, the usefulness of instructions was discussed and it was noted that some instructions in the ASCOT were skipped. Also the reference to the recall time in the instructions was mentioned. When discussing the DEMQOL, which refers to the ‘last week’, one carer noted difficulties with regard to the feelings section and stated that: “…*in his [partner’s] case, he would be both cheerful and enjoying life as well as distressed and sad and lonely – all within a fairly short time frame?”* (Carer2).

#### Proxy response

Answering the questions on behalf of a person with dementia was generally perceived as a challenging task, as “…*it’s hard to put yourself in someone else’s shoes”* (Carer, FG2) and you are “…*almost second-guessing”* (Carer, FG4). However, some carers stressed that it is “…a*ll about interpreting […] the body language”* (Carer, FG1) and knowing the person well. In this context, things that can be observed, such as mobility or personal-care of the EQ-5D-5 L were perceived as easy to answer by carers. In contrast, questions around feelings, such as those included in the DEMQOL or ICECAP-O “…*are very much subjective, and it’s difficult to honestly answer*” (Carer, FG4). Part of the problem was also that carers never talked about certain aspects of life with the person they provide care for. The challenge to answer on behalf of the person with dementia was increased if carers themselves did not easily understand the questions. Questions related to memory in the DEMQOL and QOL-AD were “…*easier to answer, as […] you’re a witness to these things”* (Carer, FG4). Despite providing clear instructions, there was a general confusion about the proxy assessments in terms of whether it should be conducted from a proxy-proxy perspective (asking proxies to rate the QoL in their proxy’s opinion) or proxy-person perspective (asking proxies to rate how they think the person with dementia would rate his/her own QoL if the person was able to communicate). After asking to what extent this would change their responses, one carer stated that she “*would just go one step more severe in most of the answers”* (Carer, FG2) if she would answer these questions from her own perspective.

## Discussion

This study used qualitative methods to further explore the content and face validity of PBMs in people with dementia and carers of people with dementia. A key focus of this study was the exploration of QoL measures based on different conceptual frameworks. The results of this study have shown that the six PBMs captured some but not all domains of QoL that were perceived as relevant. Particularly, the omission of cognition, communication, coping, and end-of-life aspects was prominent. Unsurprisingly, the most comprehensive PBM, the AQoL-8D (35 items), showed the greatest overlap with domains of QoL that were considered as important, although some redundancy was also noted, given it is a generic measures with some questions being less applicable to people living with dementia. While a lengthier questionnaire may appear favourable, our study findings also indicate that the evaluative space (i.e., what domains of quality of life are included in the questionnaire) seem to play a greater role in determining the content validity. For example, while the EQ-5D-5 L and ICECAP-O contain equal number of questions, the ICECAP-O adopts a broader evaluative space that goes beyond health-related aspects of QoL, which resulted in a greater overlap with domains of QoL that were perceived as important.

At the same time, the focus on a broader evaluative space diminished the simplicity and clarity of questions and response options. The resulting shortcomings of the ICECAP-O identified in this study are also in agreement with the study by van Leeuwen and colleagues in the Netherlands, who highlighted items of the ICECAP-O that were not always understood by older adults (Role item), double-barrelled items (Love and Friendship item), and response options that were not mutually exclusive [23]. We also identified difficulties with the capability wording in the response options of the ICECAP-O, which was noted in previous qualitative studies [62, 63]. Similar issues were observed for the ASCOT, which are in line with the study by van Leeuwen et al. in terms of the observation that instructions were often skipped and the identification of difficult and double-barrelled questions [23].

We were also able to confirm the findings from previous studies in relation to the EQ-5D (− 3 L, − 5 L) that showed that despite its overall acceptance [23, 63], it does not include attributes that are important in measuring QoL for people with dementia, such as attributes related to emotional wellbeing, social wellbeing and cognition [14]. It is noteworthy that recent research has explored the addition of a cognition dimension to the EQ-5D (EQ-5D-C), which did not necessarily perform better than the standard EQ-5D in terms of construct validity and responsiveness to change but resulted in systematically different values [64, 65]. Also the addition of a ‘dignity’ question to the EQ-5D has been previously explored [66], which has the potential to improve the sensitivity of the EQ-5D within the social-care context [67] but further research is needed.

This study found that dementia-specific measures were not always favoured over non-specific measures. The development of the descriptive classification systems of the two dementia-specific preference-based measures used psychometric analysis to attempt to cover the key areas of QoL [29, 59]. However, our study showed important omission of QoL domains in the classification systems of the DEMQOL-U (i.e., activity and self-care) and AD-5D (i.e., relationships), despite their inclusion in the DEMQOL and QOL-D, which underscores the importance of qualitative research for item selection when developing health state classification systems [68]. It is likely that these aspects might be indirectly captured by existing domains, as findings from focus groups confirming the selection of the AD-5D domains have indicated [69]. On the other hand, there were a number of QoL domains important to people with dementia and their carers that were neither included in the DEMQOL nor in the QOL-AD, which supports previous qualitative findings on the QOL-AD in terms of limitations in coverage [70]. Interestingly, the AD-5D and DEMQOL-U were also the only measures lacking items related to Autonomy. Having some levels of independence and control over life were found to be important aspects of QoL to people living with dementia. While the initial long version of the DEMQOL included an item around the ‘sense of independence’, this item was removed from the final version following the psychometric testing stage [44]. On the contrary, ‘cognition’ was exclusively measured by the AD-5D and DEMQOL-U; yet carers noted that the long list of memory-related questions in the DEMQOL may be perceived as distressing and judgemental. In line with a previous study [69], we found that cognition was considered an important domain of QoL especially for carers who observed first-hand problems with memory loss and cognitive decline in their loved ones. While people living with dementia also talked about signs of memory loss, they tended to focus more on what this meant for them in terms of their daily living and their coping mechanisms. Different perspectives (i.e., carers versus person with dementia) therefore may make for a heightened emphasis on different QoL domains. Finally, while some negative views were expressed towards items in the QOL-AD and DEMQOL that are not included in the descriptive classification systems of the AD-5D and DEMQOL-U, it is important to note that the administration of the DEMQOL and QOL-AD ultimately influences responses to items that are part of the DEMQOL-U and AD-5D.

### Selection of measure(s) and future research

Given the shortcomings in the content and face validity of existing measures, the selection of appropriate PBMs for use in economic evaluation requires careful consideration. This study could not identify one particular measure that should be recommended for future use, as the measures either lacked content validity or face validity or both. Acknowledging that the measurement of QoL is inherently subjective and that there is no gold standard, trade-offs need to be made. Our study findings have shown that when choosing one of the existing PBMs, comprehensiveness could be compromised by selecting a more feasible measure. For example, the EQ-5D-5 L was perceived as an easy and straightforward PBM but lacked content validity due to its generic nature. One option would be to complement a generic PBM with a dementia-specific PBM in an economic evaluation. Yet, our study identified omissions of important QoL domains in the DEMQOL-U and AD-5D, suggesting the need for a revised descriptive system or a new PBM. While not dementia-specific, there is currently a project underway that aims to develop a PBM for assessing the cost effectiveness of interventions across aged and social care, the Quality of Life Aged Care Consumers (QOL-ACC) [71, 72]. This PBM should be assessed further against existing dementia-specific PBMs. Complementing different PBMs in an economic evaluation also creates a challenge for decision-makers who need to consider multiple QALYs based on different PBMs. While the EQ-5D has largely dominated the field and is the preferred PBM in some countries, like the UK, it also raises the question as to which domains of QoL are considered most important for resource allocation decisions. There have been criticisms that QALYs based on the EQ-5D are too narrow and overlook the impact of health conditions and treatments on broader dimensions of QoL, especially within the context of mental health and social care [73]. This has led to the ‘Extending the QALY’ project, which aims to develop a new generic PBM of QoL for use in economic evaluation across health, social care and public health [74]. Future research to evaluate this measure in people living with dementia will be required. Finally, the importance of end-of-life aspects identified in this study also suggest the further exploration of PBMs developed in this context, such as the Palliative Care Outcome Scale [75] or the ICECAP-Supportive Care Measure [76].

Additionally, to guide further the selection of measures for future dementia research, empirical comparative analyses are warranted. This study focused upon content and face validity only but other psychometric criteria need to be assessed, including construct validity, reliability, and very importantly, responsiveness to change. In this context, another layer of validity issues will arise when considering the utility values attached to different states as described by the respective PBMs. The measures considered in this study sought exclusively preferences from the general public, except for the AQoL-8D, that included also people with mental health problems [15]. While the development of the DEMQOL and AD-5D contained a separate valuation study with carers and people with mild dementia, the final scoring algorithm is, however, based on views of the general population [28, 77, 78]. Yet, Rowen and colleagues have shown that people with dementia and carers of people with dementia gave systematically lower values than members of the general population that could impact the results of CUA and subsequent resource allocation decisions [79].

In determining an appropriate PBMs within the context of dementia, there are also a number of contextual aspects that should be considered. Although not fully explored in this study, our findings have indicated that the choice will depend on the severity of the condition, the setting, as well as the specific domains targeted by the intervention. Eliciting QoL data via PBMs may also be comprised by external factors, such as community attitudes and the role of the interviewer or the provision of assistance, which could influence people’s responses. The timing and location of data collection form additional considerations. Previous research described significant fluctuation in cognition, function, or behaviour in people with dementia, which denotes departure from usual symptom expression, often characterised as ‘good’ or ‘bad’ days [80]. This body of knowledge suggests novel administration methods, allowing for more frequent data collection to account for variability in cognitive and non-cognitive symptoms. In this context, the recall time is also relevant, as our study findings have shown that even a recall time of the past 7 days was considered by some study participants as too long due to recall problems as well as the fluctuations. Likewise, the location for the data collection must be chosen wisely to ensure an environment, where participants do not feel judged or offended, which could jeopardize the reliability and validity of responses.

Problems with cognitive function in people with dementia may compromise their ability to make judgments and give accurate responses. As such, proxy assessment to substitute or complement self-assessment is often sought. However, the reference against which one judges QoL is a key issue and the ability of the proxy to distinguish between own views and the view of the person with dementia is crucial. If taking a proxy-proxy perspective, it is important for proxies to disclose their QoL perceptions that may deviate from the patient perspective. If taking a proxy-person perspective, the agreement will highly depend on the discrepancy in expectations between the patient and the proxy. The assessment of QoL has been described as the gap between expectations and present experience [81]. Given the potential lack of reduction in expectation by proxies, it could undervalue the QoL of the person with dementia if the person was able to reduce their expectations [82]. Although this study did not use proxy-versions of the measures, it is important to clearly state the specific perspective to be taken and to examine potential validity implications. Others have proposed to provide two sets of response options for each proxy perspective [83]. In this context, it is also noteworthy that there is currently limited evidence available on the cognitive threshold beyond which it is not possible to provide a self-assessment of own QoL, which could potentially also differ between measures [84].

### Strengths and limitations

As previous qualitative studies have focused on content and face validity of a limited number of measures, a strength of our study is the exploration of six PBMs. Yet, other PBMs exist, as well as different versions of the measures explored in this study (e.g., an easy-read version of the ASCOT [85]), in addition to its proxy version that were developed for some measures, which require further validation work. While this study also included the ICECAP-O, given that it is a PBM developed for use in economic evaluation, it should be noted that it cannot be used to calculate QALYs, although a previous study has estimated capability QALYs using it [86].

Furthermore, the comparison of the content of PBMs with domains of QoL identified in this study was based on subjective judgements. It may be the case that some PBMs could capture some of the domains indirectly, which could be explored through psychometric testing. The self-report of the dementia diagnosis poses another limitation as well as the inclusion of people with mild dementia only. Nevertheless, by including carers of people with moderate and severe dementia, we were hoping to reflect the views of people with dementia across the entire severity spectrum. Due to recruitment difficulties, we were only able to recruit nine individuals living with mild dementia and may not have reached data saturation, which is the point whereby additional interviews are not expected to yield new or valuable information.

## Conclusions

The ongoing accumulation of validity evidence of PBMs for application to people with dementia and carers is essential, particularly as such measures have significant implications for the assessment of cost-effectiveness of interventions targeted for the condition. This study provided further evidence of the content and face validity of six PBMs and identified a number of aspects which may compromise the validity of data collected. Researchers should consider the advantages and disadvantages cross all measures when choosing a PBM and take the contextual aspects into account, which may favour some measures over others. Building on our findings, future qualitative studies are warranted in other settings, in addition to empirical comparative studies exploring these measures further to guide the selection of measures for future economic evaluation.

## Data Availability

In line with the ethics approval obtained, the datasets used and/or analysed during the current study cannot be made freely available.

## Abbreviations

AD-5D: Alzheimer’s Disease Five Dimensions
AQoL-8D: Assessment of Quality of Life eight dimensions measure
ASCOT: Adult Social Care Outcomes Toolkit
CDAMS: Cognitive Dementia and Memory Service
CUA: Cost-utility analysis
DEMQOL: Dementia Quality of Life measure
DEMQOL-U: Dementia Quality of Life Utility measure
EQ-5D: EuroQol five dimension measure
E-QALY: Extending the quality-adjusted life year
HRQoL: Health-related quality of life
IADL: Instrumental activities of daily living scale
ICECAP-O: ICEpop CAPability measure for Older people
QALYs: Quality-adjusted life years
QoL: Quality of life
QOL-ACC: Quality of Life Aged Care Consumers
QOL-AD: Quality of Life in Alzheimer’s Disease
PBMs: Preference-based measures
TICS: 11-item telephone interview of cognitive status
